# Natalizumab-associated progressive multifocal leukoencephalopathy

**DOI:** 10.3389/fneur.2025.1575653

**Published:** 2025-06-20

**Authors:** Trevor Glenn, Joseph R. Berger, Caleb R. S. McEntire

**Affiliations:** ^1^Department of Neurology, Mass General Brigham, Boston, MA, United States; ^2^Department of Neurology, University of Pennsylvania, Philadelphia, PA, United States

**Keywords:** progressive multifocal leukoencephalopathy, natalizumab, JC virus, relapsing remitting multiple sclerosis, demyelimating diseases

## Abstract

Progressive multifocal leukoencephalopathy (PML) is a demyelinating disease of the brain resulting from infection of oligodendrocytes by JC virus (JCV) typically occurring in association with defects of cell-mediated immunity. The clinical presentation of PML depends on its area of effect in the central nervous system and can include a broad spectrum of deficits such as focal weakness, speech difficulties, visual changes, cognitive disruptions, or ataxia. While the disease was first described in patients with B cell malignancies (Hodgkins’s lymphoma and chronic lymphocytic leukemia), a large array of immunosuppressive conditions, most notably human immunodeficiency virus, may predispose to the disorder. From 2005 on, PML was observed in patients with multiple sclerosis (MS) and Crohn’s disease being treated with natalizumab, a monoclonal antibody inhibiting α4β1 and α4β7 integrins. Risk factors for PML development were identified, and an effective risk mitigation strategy chiefly predicated on the JCV antibody index was established (an antibody index less than 0.4 is considered negative, 0.4 to 0.9 low risk, 0.9 to 1.5 medium risk, and greater than 1.5 high risk). Here we review risk stratification, diagnosis, and treatment of PML in patients receiving natalizumab.

## Introduction

Progressive multifocal leukoencephalopathy (PML) is a devastating disease of the central nervous system (CNS) that occurs most commonly in immunosuppressed patients in whom JC virus (JCV), a human polyomavirus, transforms from a latent to a more clinically active form. PML develops as a stochastic event that requires viral replication, infection of oligodendrocytes following viral genetic modification, and impaired immunosurveillance permitting viral persistence and replication within the CNS (1). Productive infection of oligodendrocytes, the myelin-producing cells of the central nervous system (CNS), results in demyelination within the brain leading to PML. More rarely, granule cells of the cerebellum are infected, leading to cerebellar degeneration in a condition known as granule cell neuronopathy. On magnetic resonance imaging (MRI), PML is often characterized by multifocal, asymmetric, T2-weighted hyperintensities with patchy diffusion restriction that can be contrast enhancing, particularly in cases of natalizumab-associated PML or after immune reconstitution has begun (2). The diagnosis can be confirmed by testing cerebrospinal fluid for JCV DNA via polymerase chain reaction when coupled with clinical and radiographic features consistent with PML or through tissue examination demonstrating the classic histopathological triad of PML (demyelination, bizarre astrocytes, and enlarged oligodendroglial nuclei) (3).

Seroepidemiological studies reveal that approximately 50–90% of adults have been infected by JCV but remain asymptomatic (4). PML most commonly develops in the context of immune suppression in patients with human immunodeficiency virus (HIV) or hematologic malignancies, and in those receiving various immunosuppressive medications that are used in rheumatologic or autoimmune conditions (5). One such medication is natalizumab, a monoclonal antibody that binds to alpha-4 integrin. The Food and Drug Administration (FDA) approved natalizumab for the treatment of relapsing–remitting multiple sclerosis (RRMS) in 2004, and the first cases of PML associated with natalizumab were recognized in 2005 (6–8). As of February 2024, the overall global incidence of natalizumab-associated PML (per Biogen) was 3.43 per 1,000 patients.

Despite the substantial risk of PML, natalizumab continues to have a role in the treatment of RRMS, as it is a highly effective medication with low rates of relapse and high rates of no evidence of disease activity (NEDA-3) status (39.0% NEDA-3 after 2 years in natalizumab compared to 22.0% in fingolimod) that are similar to the more recently introduced anti-CD20 monoclonal antibodies (9–11). Prior studies have shown similar beneficial effects on rates of relapse, MRI activity, and disability progression when comparing natalizumab and the CD-20 inhibitor ocrelizumab (12, 13). However, natalizumab is associated with risks of PML that are orders of magnitude higher than anti-CD20 monoclonal antibodies like ocrelizumab. The stratification of PML risk in patients on natalizumab is crucial for ensuring patients receive effective treatment while minimizing possible harm.

## Methods

Articles for this mini-review were primarily identified through a Pubmed search of “natalizumab and PML.” Additional resources were further found from more targeted searches for specific sections (“PML imaging” and “PML” combined with various treatments were also search terms utilized to obtain additional articles). Information was also obtained from Biogen’s website.

## Natalizumab and progressive multifocal leukoencephalopathy

PML has become increasingly associated with a variety of immunosuppressive medications, including mycophenolate mofetil, dimethyl fumarate, fingolimod, cyclosporin, cyclophosphamide, methotrexate, infliximab, and rituximab (14–17). By blocking α4β1 integrin, natalizumab prevents the entry of inflammatory cells into the CNS in a mechanism that is unique compared to other therapies used for RRMS. In patients receiving this medication, levels of CD4 + and CD8 + T cells as well as CD19 + and CD138 + positive B cells are significantly reduced in cerebrospinal fluid (CSF), and CD4/CD8 ratios in the CSF are similar to those who are infected with HIV (18). While this immunomodulatory effect can be beneficial in reducing autoimmune damage in patients with RRMS, it may also predispose to certain infections, particularly PML, though the precise mechanisms underlying this risk remain uncertain. Currently, it is believed that those most important are its ability to enhance the likelihood of transformation of the archetype virus to the prototype (neurotropic) virus, to increase expression of JCV within CD34 lymphocytes (19), and to limit the ability of JCV-specific T lymphocytes to clear the infection from the brain (20).

Studies have since been conducted to help better evaluate the risk of PML in patients on natalizumab, specifically looking at duration of therapy and JCV antibody status prior to and while on treatment. One such study of 3,180 patients examined JCV status and seroconversion in patients with RRMS who were receiving natalizumab. Prior to treatment, 56.3% of patients were JCV positive. Of the patients who were initially JCV negative, 10.9% became JCV positive during the course of their treatment (21). JCV serostatus can be quantified into a JCV index, which calculates levels of anti-JCV antibodies in the serum to classify patients into the following categories: Negative (less than 0.4), low risk (0.4 to 0.9), medium risk (0.9 to 1.5), and high risk (greater than 1.5). One study evaluated how the JCV index changed over time for 525 patients being treated with natalizumab. They found that patients’ JCV indices tended to increase with duration of therapy: 20 patients went from negative to positive, the medium risk group grew from 24 to 31, and the high risk group went from 117 to 131 (21).

JCV serostatus is crucial in assessing a patient’s risk of PML. A negative JCV serostatus suggests that the individual has not been exposed to the virus and is at no risk of PML, although a false negative rate of 2.5% has been reported (22). However, the frequency with which individuals convert from negative to positive status when serially determined suggests that there may simply be a low level of antibody production in a previously infected person and that viral replication allowed by immunosuppression has resulted in an anamnestic response that increases antibody production sufficiently to allow detection. There is a clear association between JCV index and PML; a prior study found a difference in JCV index levels in those treated with natalizumab who did not develop PML (average JCV index of 1.4) compared to those who did develop PML (average JCV index of 2.4) (23). Other studies estimate that, for patients on natalizumab, a negative JCV antibody has an associated PML risk of 1 in 10,000, a JCV index that is positive but less than 1.5 has a risk of 1 in 5,882, and an index of greater than 1.5 has a risk of 1 in 855. The risk continues to increase the longer a patient is receiving the medication; after 24–48 months of being on the medication, an index of greater than 1.5 is associated with a 1/113 risk of developing PML (24).

A risk-stratification table of risk for PML in patients receiving natalizumab has been provided by Biogen (64) based on exposure time to the medication, antibody index value, and prior immunosuppressant use. For this reason, it has been recommended to check an anti-JCV antibody approximately every 6 months while on natalizumab (24) and discontinue the drug when levels exceed 1.5. Additionally, prior studies have shown that the use of extended interval dosing at 5–6 week intervals was associated with a reduced risk of PML (25). Alternatively, the risk of PML can be virtually eliminated by confining natalizumab treatment to patients who are JCV seronegative and remain so, regardless of the level. In 2023, the FDA approved the first biosimilar to natalizumab (known as natalizumab-sztn) for use in RRMS. To go along with the new biosimilar, a new JCV index test was developed (as the standard Stratify platform that is offered by Biogen is only available to those on natalizumab and not the biosimilar). This is known as the Immunowell platform, and it is also an enzyme-linked immunosorbent assays (ELISA) test. A recent study evaluated JCV indices in a cohort of 39 patients with MS as they transitioned from natalizumab to natalizumab-sztn (26). They found that JCV antibody levels increased from 13% (via the Stratify platform) to 52% (via the Immunowell platform), which may be related to differences in the tests rather than an increase in JCV antibody (JCV indices were only tested with the Immunowell platform after the patients had switched to the biosimilar in this cohort). However, another recent study compared Stratify and Immunowell JCV indices on patients who were receiving natalizumab and found an 85.5% agreement between the two tests, with the Immunowell platform tending to categorize patients as higher risk (27).

If a clinician elects to treat a patient who is JCV seropositive with natalizumab, more frequent MRI monitoring (every 3–4 months) is recommended to determine whether PML has developed (28). Brain MRI may detect PML in a presymptomatic phase (24), which can lead to earlier intervention (cessation of immunosuppression) and improved outcomes. One challenge with presymptomatic PML detection is distinguishing it from RRMS disease progression. Some studies have found certain radiologic patterns may help differentiate these disease entities. For instance, the punctate pattern or “Milky Way sign” refers to the appearance of multiple punctate areas of T2-weighted hyperintensities surrounding the main component of a new lesion (29). One study that compared 20 patients with PML (14 of which were associated with natalizumab and 9 of which were presymptomatic) to 80 patients without PML but with multiple sclerosis found that the punctate pattern was found in 18 of the patients with PML (including all 14 associated with Natalizumab) and in none of the patients without PML (30). Thus, the appearance of the punctate pattern may be more indicative of PML than RRMS activity. Other factors on MRI that may be suggestive of PML rather than RRMS activity include a more irregular shape with ill-defined border toward the white matter and being less hypointense on T1-weighted images compared to what is expected for acute lesions due to multiple sclerosis (24). Solitary lesions that are periventricular are more associated with multiple sclerosis activity (29).

## Management of natalizumab-associated progressive multifocal leukoencephalopathy

While there are ways to help predict risk of PML in patients receiving natalizumab, cases of natalizumab-associated PML continue to occur. Prior studies have found survival rates as high as, or higher than, 70%. One study that followed 15 patients for an average of 21 months had no fatalities, although patients can have significant and lasting disability (31–34). Younger age at diagnosis, better functional status prior to diagnosis, lower JC viral load, and more localized brain involvement at time of diagnosis have all been associated with improved survival (24). Patients who are asymptomatic at time of diagnosis also have improved functional outcomes and survival (35).

Natalizumab should be discontinued in a patient who develops PML while receiving the medication. However, because the medication’s immunosuppressive effects can persist even up to 12 weeks after the last administration, plasma exchange or immunoadsorption have been used in an attempt to more quickly wash out the monoclonal antibody to expedite immune reconstitution (36). While it has previously been demonstrated that plasma exchange accelerate natalizumab clearance (37), whether this translates to clinical benefit remains uncertain. A study that followed 15 patients with natalizumab-associated PML treated with plasma exchange or immunoadsorption reported no mortalities after 21 months, although as all patients received interventions and survived, no inter-group comparisons for treatment or survival could be made (33). A larger study evaluated 219 patients with natalizumab-associated PML where 84% underwent plasma exchange, and, after a mean follow-up time of 11.5 months, there was no difference in survival or clinical outcomes compared to those who did not receive plasma exchange (38). Another, smaller study of 42 patients with natalizumab-associated PML found similar results of plasma exchange not providing a significant benefit after a 12-month follow-up period (39). While undergoing plasma exchange to expedite natalizumab clearance mechanistically makes sense, more evidence is needed to better determine its clinical utility for natalizumab-associated PML. The implementation of either plasma exchange or immunoadsorption is probably best reserved for individuals who have received their natalizumab within 3 weeks of diagnosis as the half-life of natalizumab is about 11 days in adults.

The single best treatment of PML is the restoration of the immune system. Fortunately, in natalizumab-associated PML, removing the offending agent can return the immune system to its baseline state. To date, no adjunctive therapy has been demonstrated to be effective in treating PML in a randomized, controlled trial (although additional trials are underway). Among the adjunctive therapies that have been explored are medications such as cytarabine, topotecan, mirtazapine, and mefloquine that mainly target JCV replication or entry into host cells (see Table 1 for a summary of therapies that have been evaluated for efficacy in the treatment of PML) (40, 41). In a phase II trial, the largest clinical trial of PML treatment to date, cytarabine, though effective in preventing viral replication *in vitro*, had no effect on PML survival in HIV-associated PML (42–44). Another trial investigated topotecan in a small series of patients with HIV-associated PML as topoisomerase inhibitors had previously been shown to have in vitro efficacy against JCV replication (45). This trial was terminated early without a substantial beneficial response (46). Mirtazapine, by blocking 5-HT2A receptors, appears to block entry of JCV into astrocytes in vitro (47) resulting in its use and occasional case reports documenting symptomatic improvement after its initiation (48–50). However, no large-scale studies have shown substantial benefit. Mefloquine, an anti-malarial that is thought to target the 80S ribosome of Plasmodium falciparum, has been shown in some case reports to also result in symptomatic improvement in patients with PML (51). However, other studies have found no benefit from mefloquine administration (52), and some differences in response may be mediated by genetic polymorphisms in the p-glycoprotein transporter, which can affect the drug’s absorption and elimination (53). Granulocyte-colony stimulating factor G-CSF (known as filgrastim), which has been used to help restore immune system function after chemotherapy, has also previously been investigated to see if confers a benefit to patients with natalizumab-associated PML, as it may accelerate the development of IRIS to more effectively clear JCV (54). Patients who received filgrastim did develop IRIS more rapidly than has generally been described in the literature, suggesting that filgrastim did help promote immune reconstitution. However, IRIS carries its own dangers such as increased risk of seizure and potentially fatal cerebral edema.

**Table 1:** Prior therapies explored for the treatment of progressive multifocal leukoencephalopathy.

Therapy	Mechanism of action	Arguments for efficacy	Arguments against efficacy or if current trials are ongoing
Plasma exchange	Removal of immunosuppressive agent	Dahlhaus et al. (33) (no mortality after 21 months in 15 patients)	Landi et al. (38) (no improvement in survival or outcomes) Scarpazza et al. (39) (no symptomatic benefit after 12 months)
Cytarabine	Pyrimidine analogue that inhibits alpha-DNA polymerase and inhibits DNA repair	Hou and Major (43) (*in vitro* data, inhibits JCV replication)	Hall et al. (42) (no effect on survival)
Topotecan	Topoisomerase I inhibitor	Kerr et al. (45) (in vitro data, inhibits JCV replication)	Royal et al. (46) (trial terminated early without substantial beneficial response)
Mirtazapine	Blocks 5-HT2A receptors	Elphick et al. (47) (in vitro, blocks JCV entry into astrocytes) Cettomai and McArthur (49) (case series showing symptomatic or radiographic improvement) Mullins et al. (50) (case report showing symptomatic improvement) Alwehaibi et al. (48) (case report documenting symptomatic improvement)	Evidence is anecdotal; no trials have been performed
Mefloquine	Targets the 80S ribosome of Plasmodium falciparum	Shin et al. (51) (case report showing symptomatic improvement)	Clifford et al. (52) (no benefit found in a randomized controlled trial)
Filgrastim	Granulocyte colony stimulating factor	Stefoski et al. (54) (7/17 patients recovered near to baseline)	Increases risk of IRIS; no trials have been performed
Pembrolizumab	Blocks programmed cell death protein 1	Cortese et al. (55) (clinical improvement in 5/8 patients) Beudel et al. (65) (clinical improvement in 2/2 patients)	Pawlitzki et al. (56) (case report of a patient continuing to decline) Trials are ongoing
Virus-Specific T Cells	Infusion of cells to help recognize and eliminate the target pathogen	Muftuoglu et al. (57) (cases series showing symptoms stabilized) Möhn et al. (58) (case series showing stabilization or improvement in symptoms)	Trials are ongoing
Interleukin 7	Cytokine that plays a role in T cell activation	Lajaunie et al. (59) (survival benefit in some patients out of a cohort of 64, specifically in those with PML secondary to underlying hematologic malignancies) Miskin et al. (60) (case report showed survival benefit)	Trials are ongoing
Interleukin 2	Cytokine that plays a role in T cell activation	Buckanovich et al. (61) (case report showing improvement in symptoms) Kunschner and Scott (62) (case report showing improvement in symptoms) Przepiorka et al. (63) (case report showing improvement in symptoms)	Evidence is anecdotal; no trials have been performed

IRIS, Immune reconstitution inflammatory syndrome.

As restoration of the immune system appears fundamental to PML outcome, attempts have also been made to increase neuroimmunosurveillance. One strategy employs the use of checkpoint inhibitors. A small trial of pembrolizumab (which blocks programmed cell death protein 1) in 8 patients with PML secondary to a variety of causes suggested improvement in some patients (55). However, mixed results from pembrolizumab have also been reported in case reports (56). There are also ongoing trials evaluating the efficacy of cell-based therapies for PML. For instance, the use of allogenic BK virus-specific T cell infusions for the treatment of PML has previously been shown in case reports to have a beneficial effect for patients with PML. Given the genetic similarities between the polyomaviruses BK and JC viruses, T cells that had been developed against the BK virus were hypothesized to also be effective against JCV. Three patients with PML were infused with these T cells. Two improved symptomatically and radiographically, and cleared JCV from their CSF, while the other patient had a stabilization of symptoms and reduction in JCV viral load (57). Additionally, the use of virus-specific T-cells from partially human leukocyte antigen (HLA) matched donors has been shown to result in clinical stabilization or improvement of symptoms with reduction of viral load in 22 patients (from a cohort of 28 who received the virus-specific T-cells) (58).

Trials are also underway for the use of recombinant interleukins (IL) in PML. Studies of IL-7, which promotes T cell function, found that IL-7 may confer a survival benefit but in PML secondary to hematologic malignancies and post-transplant immunosuppression patients but not underlying HIV (59, 60). IL-2 has been demonstrated in case reports to have benefit in patients with PML; however, data with larger cohorts or in trials are lacking (61–63).

## Clinical summary

The use of natalizumab in the treatment of RRMS leads to an increased risk of developing PML. Duration of treatment and JCV antibody seropositivity (and specifically antibody index) can be used to risk-stratify patients on natalizumab and allow for more individualized conversations regarding benefits and potential hazards of therapy. Tracking the JCV antibody index while on treatment and monitoring for the possible development of PML with serial MRI scans can help further personalize a patient’s possible risk of PML, as well as potentially capture PML in the presymptomatic stage, thus leading to improved outcomes. While RRMS disease activity can appear similar to PML lesions on MRI, certain features have been identified (such as the punctate pattern or “Milky Way” sign) that are more closely associated with PML.

If a patient develops PML while on natalizumab, immunosuppressive therapy should be discontinued. The benefit of plasma exchange is unclear. Other therapies such as mirtazapine, mefloquine, or pembrolizumab have been evaluated for possible benefit in patients with PML with mixed results or slight symptomatic improvement, and trials in interleukin- or cell-based therapies are ongoing. The most effective current management paradigm for natalizumab-associated PML is to screen patients for JCV antibody index to avoid development of the disease and to discontinue the therapy if any signs of PML development are detected.
